# Inorganic nitrate and nitrite supplementation fails to improve skeletal muscle mitochondrial efficiency in mice and humans

**DOI:** 10.1093/ajcn/nqz245

**Published:** 2019-10-10

**Authors:** Maria Ntessalen, Nathan E K Procter, Konstantin Schwarz, Brodie L Loudon, Magdalena Minnion, Bernadette O Fernandez, Vassilios S Vassiliou, David Vauzour, Melanie Madhani, Dumitru Constantin‐Teodosiu, John D Horowitz, Martin Feelisch, Dana Dawson, Paul G Crichton, Michael P Frenneaux

**Affiliations:** 1 Institute of Medical Sciences, University of Aberdeen, Aberdeen, United Kingdom; 2 Norwich Medical School, University of East Anglia, Norwich, United Kingdom; 3 Clinical & Experimental Sciences, Faculty of Medicine, University of Southampton, Southampton General Hospital, Southampton, United Kingdom; 4 Institute of Cardiovascular Sciences, University of Birmingham, Birmingham, United Kingdom; 5 Medical Research Council/Arthritis Research UK Centre for Musculoskeletal Ageing Research, National Institute for Health Research Nottingham Biomedical Research Centre, School of Life Sciences, Nottingham University Medical School, Nottingham, United Kingdom; 6 Department of Cardiology, The Queen Elizabeth Hospital, University of Adelaide, Adelaide, South Australia, Australia; 7 Department of Cardiology, School of Medicine & Dentistry, University of Aberdeen, Aberdeen, United Kingdom

**Keywords:** nitrate, nitrite, mitochondria, uncoupling proteins, pyruvate dehydrogenase

## Abstract

**Background:**

Inorganic nitrate, abundant in leafy green vegetables and beetroot, is thought to have protective health benefits. Adherence to a Mediterranean diet reduces the incidence and severity of coronary artery disease, whereas supplementation with nitrate can improve submaximal exercise performance. Once ingested, oral commensal bacteria may reduce nitrate to nitrite, which may subsequently be reduced to nitric oxide during conditions of hypoxia and in the presence of “nitrite reductases” such as heme- and molybdenum-containing enzymes.

**Objective:**

We aimed to explore the putative effects of inorganic nitrate and nitrite on mitochondrial function in skeletal muscle.

**Methods:**

Mice were subjected to a nitrate/nitrite-depleted diet for 2 wk, then supplemented with sodium nitrate, sodium nitrite, or sodium chloride (1 g/L) in drinking water ad libitum for 7 d before killing. Skeletal muscle mitochondrial function and expression of uncoupling protein (UCP) 3, ADP/ATP carrier protein (AAC) 1 and AAC2, and pyruvate dehydrogenase (PDH) were assessed by respirometry and Western blotting. Studies were also undertaken in human skeletal muscle biopsies from a cohort of coronary artery bypass graft patients treated with either sodium nitrite (30-min infusion of 10 μmol/min) or vehicle [0.9% (wt:vol) saline] 24 h before surgery.

**Results:**

Neither sodium nitrate nor sodium nitrite supplementation altered mitochondrial coupling efficiency in murine skeletal muscle, and expression of UCP3, AAC1, or AAC2, and PDH phosphorylation status did not differ between the nitrite and saline groups. Similar results were observed in human samples.

**Conclusions:**

Sodium nitrite failed to improve mitochondrial metabolic efficiency, rendering this mechanism implausible for the purported exercise benefits of dietary nitrate supplementation. This trial was registered at clinicaltrials.gov as NCT04001283.

## Introduction

Recent data suggest that there are considerable health benefits (particularly cardiovascular) from diets high in inorganic nitrate (${\rm{NO}}_3^ - $). The nitrate (${\rm{NO}}_3^ - $)–nitrite (${\rm{NO}}_2^ - $)–nitric oxide (NO) pathway, whereby dietary inorganic nitrate is processed via the entero-salivary circuit to release ${\rm{NO}}_2^ - $ and NO (1), is attracting considerable interest. Adherence to a Mediterranean-style diet (typically rich in dietary nitrates) is associated with a lower risk of developing coronary artery disease or heart failure (2, 3). In healthy individuals, increased dietary nitrate consumption has been shown to reduce the oxygen cost of skeletal muscle work during submaximal exercise, implying increased metabolic efficiency (4-8); in clinical settings, a regime of dietary nitrate supplementation improved exercise performance in heart failure with preserved ejection fraction (HFpEF) and muscle contractile function in heart failure with reduced ejection fraction (HFrEF) (9, 10). However, each of these studies was limited by their small sample size. The recent, comparatively large, INDIE (Inorganic Nitrite Delivery to Improve Exercise Capacity) trial (utilizing inhaled nitrite) showed no benefit on exercise capacity in HFpEF (11). It is unclear whether the smaller studies were false positives, or whether the shorter plasma half-life of the inhaled nitrite approach failed to work because of the pulsatile rather than sustained nature of elevation of nitrite.

Oral inorganic nitrate ingestion has also been documented to lower blood pressure (5, 12-14), and nitrite has been shown to inhibit platelet aggregation (15, 16), promote vasodilation (17), ameliorate pulmonary hypertension (18-20), and attenuate cardiac ischemia-reperfusion injury in experimental models (21, 22).

The physiological mechanisms underlying these apparent improvements in metabolic efficiency are not well understood. It has been proposed that ingestion of inorganic nitrate can improve mitochondrial coupling efficiency (i.e., the relationship between mitochondrial respiration and ATP generation) via reduced expression of proteins believed to facilitate mitochondrial proton leak [e.g., the ADP/ATP carrier proteins (AACs) and uncoupling proteins (UCPs)] (6). However, recent work has cast doubt on these findings. Whitfield et al. (23) observed a reduction in whole body oxygen consumption in response to beetroot juice with no alteration in mitochondrial coupling efficiency (23). Similarly, Monaco et al. (24) demonstrated improved hemodynamics with no change in cardiac mitochondrial coupling efficiency in response to oral sodium nitrate (24). An alternative mechanism would entail a shift in the Randle cycle [see Hue and Taegtmeyer (25) for a review] away from fatty acids toward glucose utilization with increased coupling of glycolysis to carbohydrate oxidation, which would be accompanied by increased efficiency with respect to oxygen consumption. Previous work has reported that inorganic nitrate is capable of increasing skeletal muscle glucose utilization in the context of type 2 diabetes (26, 27).

The aforementioned studies have largely focused on investigating sodium nitrate supplementation, as opposed to sodium nitrite, the putative “active principle” of inorganic nitrate. Given the limitations of entero-salivary conversion of sodium nitrate to sodium nitrite, the following investigation sought to elucidate the molecular mechanisms whereby sodium nitrite may improve metabolic efficiency in skeletal muscle.

## Methods

### Treatment conditions

Male C57/bl6 mice, 3–5 mo old, were obtained from Harlan Laboratory UK Ltd and the University of East Anglia Disease Modelling Unit. After a 2-wk acclimatization period to local vivarium conditions, mice were subjected to a nitrate/nitrite-depleted diet for 2 wk. Mice were then administered either sodium chloride, sodium nitrate, or sodium nitrite at a concentration of 1 g/L in drinking water ad libitum for 7 d. All experimental procedures and protocols used in this study were reviewed and approved by the Animal Welfare and Ethical Review Body and were conducted according to the specifications of the United Kingdom Animals (Scientific Procedures) Act, 1986.

### Determination of plasma nitrate/nitrite concentrations

Mice were anaesthetized using 5% isoflurane and 4 L/min oxygen, and blood was collected via intracardiac puncture into EDTA-containing anticoagulant tubes containing 100 µL 10 mM N-ethylmaleimide. Plasma was generated by centrifugation at 2000 × *g* for 10 min at room temperature, then snap-frozen using liquid nitrogen and stored at −80°C until analysis. Plasma concentrations of nitrate and nitrite were determined by a dedicated HPLC system (ENO-20; Eicom), as previously reported (28).

### Mitochondrial isolation

A subsequent cohort of mice (C57/bl6 mice, 3–5 mo old) were killed by cervical dislocation and gastrocnemius muscle samples harvested from both hind limbs were finely dissected in 1 mL cold isolation buffer (100 mM sucrose, 100 mM KCl, 50 mM Tris-HCL, 1 mM KH_2_PO_4_, 0.1 mM EDTA, and 0.2% BSA; pH 7.2) and washed, before incubating with 12 U/g protease (Sigma-Aldrich) in 5 mL isolation buffer for 2 min on ice. The tissue was removed from the protease by brief centrifugation (200 × *g* for 5 min at 4°C) and resuspended in 20 mL fresh isolation buffer. The suspension was then transferred into a Dounce homogenizer and homogenized using 10 up-and-down strokes on ice, before centrifugation at 700 × *g* for 10 min at 4°C. The supernatants were filtered (70 µm cell strainer) and centrifuged at 8000 × *g* for 10 min at 4°C. The pellets were resuspended in cold isolation buffer and centrifuged at 7000 × *g* for 10 min at 4°C. Isolated mitochondria were resuspended in 150 µL suspension buffer (225 mM mannitol, 75 mM sucrose, 10 mM Tris, and 0.1 mM EDTA; pH 7.2), assayed for protein content (Pierce BCA Protein Assay Kit; ThermoFisher Scientific), and kept on ice until further use.

### Mitochondrial respiration

#### Seahorse XF24 analyzer

The oxygen consumption rate (OCR) of mitochondria isolated from murine skeletal muscle was initially measured using an XF24 analyzer (Seahorse Bioscience). The mitochondrial suspension was deposited onto XF24 cell culture microplates (Seahorse Bioscience) at a concentration of 2.5 µg/50 µL in mitochondrial assay solution (MAS) buffer [70 mM sucrose, 220 mM mannitol, 10 mM KH_2_PO_4_, 5 mM MgCl_2_, 2 mM HEPES, 1.0 mM EGTA, and 0.2% (wt:vol) fatty acid–free BSA; pH 7.2] containing 10 mM pyruvate/5 mM malate (mitochondrial complex I substrate). The plates were centrifuged at 2000 × *g* for 20 min at 4°C. Prewarmed (37°C) MAS buffer containing complex I substrate was added to the equivalent wells to a final volume of 495 µL. The plate was equilibrated in a carbon dioxide–free incubator for 8 min at 37°C, then placed in the analyzer. OCR was measured in triplicate at baseline, and after sequential addition of 4 mM ADP, 2.5 µg/µL oligomycin, 4 µM carbonyl cyanide-p-trifluoromethoxyphenylhydrazone (FCCP), and 4 µM antimycin A (mitochondrial “stress test”) according to the manufacturer's instructions. The OCRs were used to determine maximal mitochondrial respiration (FCCP-induced OCR minus antimycin A OCR), leak (oligomycin-induced OCR minus antimycin A OCR), and the respiratory control ratios (ADP-induced OCR:oligomycin-induced OCR), accordingly.

#### Oxygen electrode

For a more detailed analysis of respiratory function, mitochondrial oxygen uptake activity was measured using a Clark-type oxygen electrode (Oxytherm; Hansatech), calibrated following the manufacturer's instructions. Aliquots (0.75 mg) of isolated muscle mitochondria were suspended in 1.5 mL air-saturated MAS buffer at 37°C with the addition of 10 mM pyruvate/5 mM malate to initiate mitochondrial oxygen uptake activity (at an acquisition rate of 5 Hz, stirring at 27 rpm). For the assessment of key respiratory parameters, OCRs were measured after the sequential addition of 4 mM ADP (to initiate maximal “state 3” respiration), 5 µM oligomycin (to measure the rate associated with proton leak), 7 µM carboxyatractyloside (to determine the contribution of AAC to the proton leak rate), 2 µM FCCP (to give the maximal uncoupled activity), and 2 µg/mL antimycin A (to fully inhibit mitochondrial respiration). Alternatively, 2 sequential additions of 400 nmol ADP (ε_259_ = 15,400 M^−1^/cm) were used to induce transient state 3 rates for phosphate:oxygen (P:O) ratio determination. The corresponding amount of oxygen consumed during each state 3 burst was estimated as described in Brown and Cooper (29). Importantly, the order in which “treated” and “control” samples were measured in the electrode was alternated each day to avoid any time-dependent effects (e.g., uncoupling) inadvertently being ascribed to one mitochondrial sample type over the other.

### Immunoblotting

#### Mouse tissue

Mice were killed by cervical dislocation. Gastrocnemius muscle was harvested, snap-frozen in liquid nitrogen, then stored at −80°C. Tissue samples were homogenized in lysis buffer (100 mM Tris-HCl, 2 mM Na_3_VO_4_, and 5 mM NaF; pH 7.4) using a mortar and pestle. Homogenate was subject to 3 freeze–thaw cycles using liquid nitrogen, then centrifuged at 14,000 × *g* for 15 min at 4°C. Supernatants were retained and stored at −20°C. Total extracted protein was determined by Bradford assay.

SDS-PAGE was conducted under reducing conditions. Samples were loaded in Laemmli buffer containing β-mercaptoethanol, at 20 µg total protein per lane in 10% acrylamide gels. Electrophoresis was conducted at 60 V for 20 min, then 120 V for 80 min. Separated proteins were transferred onto 0.2 µm polyvinylidene difluoride (PVDF) membrane (Bio-Rad) at 100 V for 75 min. Membranes were blocked in 5% (wt:vol) commercially available fat-free milk in 0.1% (vol:vol) Tris-buffered saline (TBS)-Tween for 1 h at room temperature. All antibodies were diluted using 5% (wt:vol) fat-free milk in 0.1% (vol:vol) TBS-Tween. Primary antibodies (Uncoupling Protein 3, 1:500 dilution, Abcam; Pan-Adenine Nucleotide Translocase, 1:500 dilution, Santa-Cruz) were incubated overnight at 4°C, followed by incubation with anti-rabbit or anti-goat horseradish peroxidase–conjugated secondary antibodies at 1:5000 dilution (Cell Signaling Technology) for 1 h at room temperature. Proteins were visualized using chemiluminescent substrate (ECL:2.5 mM luminol, 0.4 mM coumaric acid, hydrogen peroxide) and imaged in a Fusion SL imager (Peqlab). After visualization, membranes were stained with 0.2% (wt:vol) Coomassie blue for normalization to the amount of protein per lane (30). Images were analyzed using ImageJ (NIH).

#### Human tissue

**FIGURE 1:**
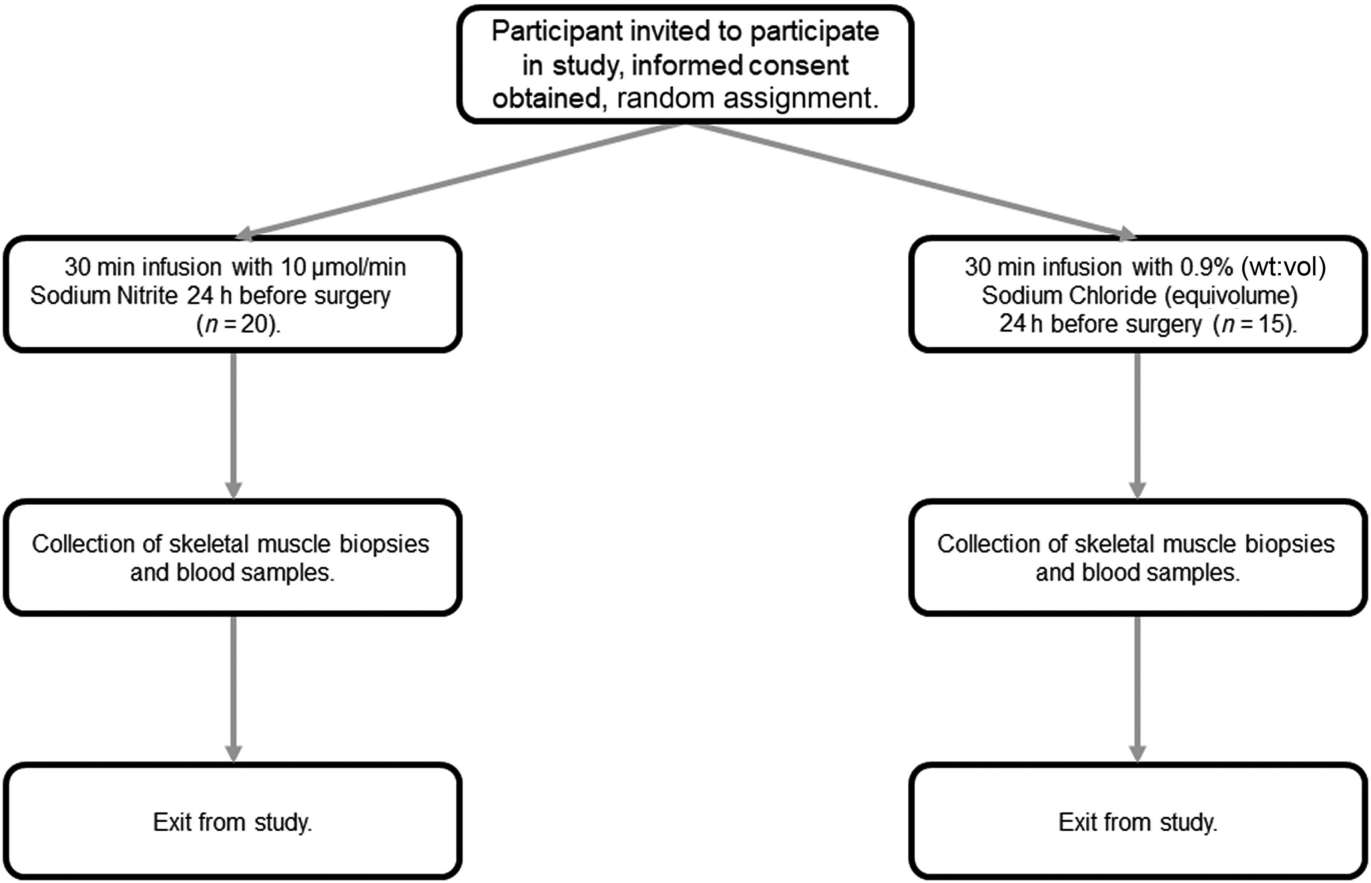
A schematic of the trial design (acute phase) for the “Effect of Nitrite on Cardiac Muscle and Blood Vessels in Patients Undergoing Coronary Artery Bypass Grafting Surgery” study.

Participants were recruited into the “Effect of Nitrite on Cardiac Muscle and Blood Vessels in Patients Undergoing Coronary Artery Bypass Grafting Surgery” study (NCT04001283), a randomized, placebo-controlled, double-blind clinical study investigating the potential mechanisms underlying sodium nitrite as a medical therapy (**Figure 1**). Eligible participants were patients undergoing coronary artery bypass graft (CABG) surgery, with reasons for exclusion being inability to provide informed consent, pregnancy or of child-bearing potential, type 1 diabetes mellitus, New York Heart Association class III–IV heart failure with left ventricular ejection fraction <40%, renal impairment requiring dialysis, and unstable coronary syndrome (occurring within the preceding 2 wk). Study participants were randomly assigned into either the treatment (sodium nitrite 10 μmol/min infused over a period of 30 min) or control [0.9% (wt:vol) saline–infused equivolume over a period of 30 min] arms, with blood samples collected immediately before and after infusion, then 6 h and 24 h postinfusion, and at time of biopsy. Infusion occurred 24 h before scheduled surgery. Skeletal muscle (pectoral muscle), right atrial appendage, and left ventricular biopsies were collected immediately before aortic cross-clamping, snap-frozen using liquid nitrogen, and stored at −80°C until analysis. The study was approved by the institutional Ethics of Human Research Committee and complied with the Declaration of Helsinki. Written informed consent was obtained in all cases.

Samples were snap-frozen in liquid nitrogen, after which 100–150 µL NP40 cell lysis buffer (ThermoFisher Scientific) containing a Halt protease and phosphatase inhibitor cocktail (ThermoFisher Scientific) was added. Samples were manually crushed, then homogenized on ice using a Pellet Pestle (Sigma-Aldrich). Samples were then snap-frozen using liquid nitrogen and allowed to thaw on ice, after which they were homogenized for a second time. Samples then underwent a further 2 freeze–thaw cycles using liquid nitrogen, mixing on a vortex after thawing each time. Samples then underwent centrifugation at 16,700 × *g* for 10 min at 4°C; the supernatants were collected and stored at −80°C. Protein determination was performed using the Bio-Rad DC Assay (Bio-Rad). Samples were loaded into Laemmli buffer at a concentration of 1.5 mg/mL and underwent SDS-PAGE using 10% acrylamide gels under reducing conditions for ∼1 h at 0.08 A, after which they were transferred onto PVDF membrane (GE Healthcare) for ∼1.5 h at 0.38 A. Membranes were subsequently blocked for a minimum of 2 h in either 5% (wt:vol) BSA or skim milk in TBS-Tween, as appropriate. Primary antibody targets were Uncoupling Protein-3 (1:1000, Abcam), Adenine Nucleotide Translocase 1 (1:1000, Abcam), Adenine Nucleotide Translocase 2 (1:1000, Cell Signaling Technology), Phospho(serine 232)-PDH (1:2000, Calbiochem), Phospho(serine 293)-PDH (1:1000, Abcam), Phospho(serine 300)-PDH (1:1000, Calbiochem), PDH (1:1000, Cell Signaling Technology), and Vinculin (1:1000, Abcam). Secondary detection was done using horseradish peroxidase–conjugated goat anti-rabbit (1:1000, Cell Signaling Technology) or goat anti-mouse (1:1500, Dako) antibodies. Membranes were developed using Pierce ECL western blotting substrate (ThermoFisher Scientific) and images captured using a ChemiDoc-It^2^ imager (Ultra-Violet Products Ltd) with VisionWorksLS 8.1.2 software (Ultra-Violet Products Ltd). Images were analyzed using ImageJ and normalized to Vinculin expression.

### Pyruvate dehydrogenase activity

The activity of the active form (i.e., dephosphorylated) of pyruvate dehydrogenase (PDH) was determined through acetyl-CoA formation, measured after condensation with radioactive oxaloacetate to form citrate, as previously reported (31, 32).

### Statistics

All data are expressed as mean ± SD unless stated otherwise. Differences between groups were analyzed by independent *t* test or ANOVA (as appropriate) for parametric data or Mann–Whitney *U* test for nonparametric data. Frequency distributions across treatment groups were assessed by χ^2^ test. The human studies were powered in order to detect a treatment difference of 20% in expression and phosphorylation status of metabolic proteins (i.e., PDH, UCP3, AAC1, and AAC2), as determined by immunoblotting. Based on an assumed CV of 20%, *n* = 14 patients per group would be required for 80% confidence at a 2-sided α of 0.05. Similarly, in murine experiments a treatment difference of 15% in leak respiration (oligomycin respiration minus antimycin A respiration) would require *n* = 8 per group for 80% confidence at a 2-sided α of 0.05. Analyses were performed using GraphPad Prism version 7.01 or IBM SPSS Statistics version 25.0.0.1.

## Results

### Murine uptake of supplemented sodium nitrate or nitrite

**FIGURE 2:**
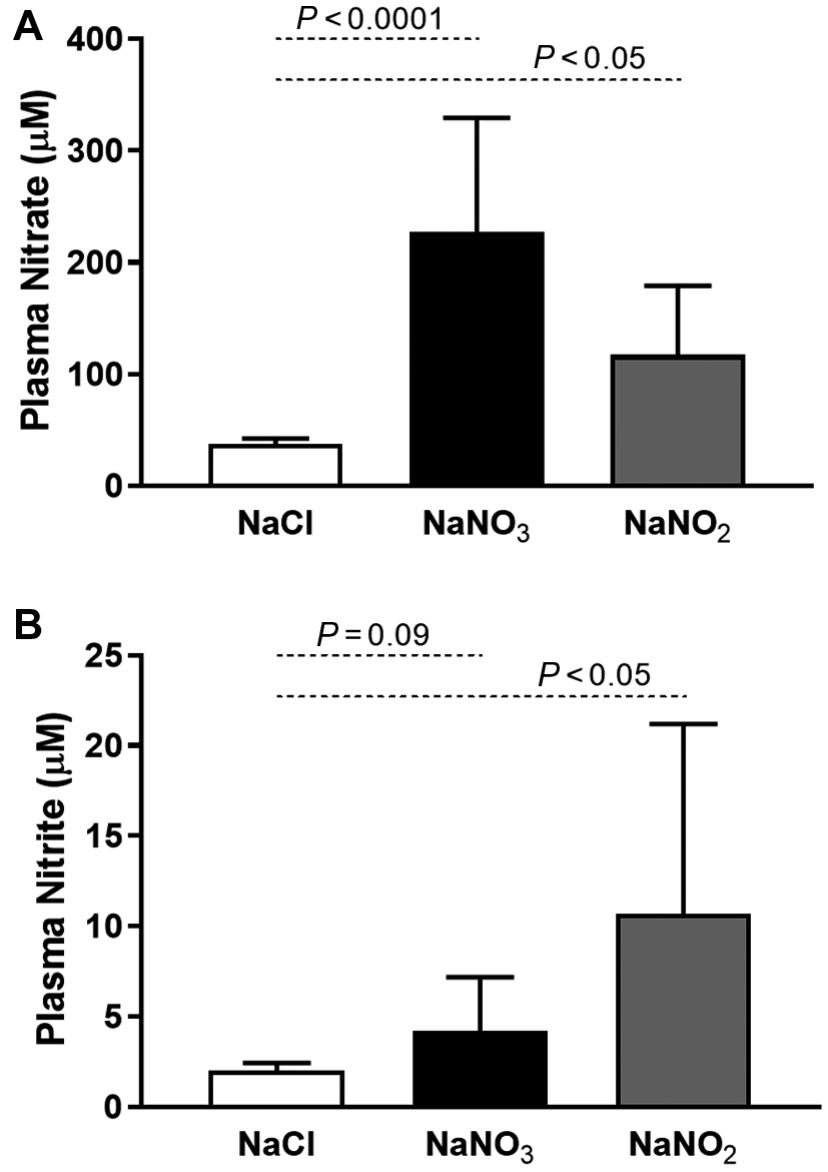
Plasma ${\rm{NO}}_3^ - $ and ${\rm{NO}}_2^ - $ concentrations in mice after supplementation with sodium nitrate (*n* = 10–13 per group) or sodium nitrite (*n* = 6 per group) for 7 d. (A) Plasma ${\rm{NO}}_3^ - $ concentrations in mice supplemented with sodium nitrate or sodium nitrite. (B) Plasma ${\rm{NO}}_2^ - $ concentrations in mice supplemented with sodium nitrate or sodium nitrite.

Plasma concentrations of ${\rm{NO}}_3^ - $ and ${\rm{NO}}_2^ - $ after 7 d supplementation of mice with sodium nitrate or sodium nitrite are depicted in **Figure 2**. Supplementation with sodium nitrate resulted in an ∼7-fold increase in plasma ${\rm{NO}}_3^ - $ concentrations (227 ± 103 µM compared with 33 ± 10 µM, *P* < 0.0001), with a nonsignificant trend toward increased plasma ${\rm{NO}}_2^ - $ concentrations (4 ± 3 µM compared with 2 ± 1 µM, *P* = 0.09). This result may be indicative of a limited entero-salivary circuit for reduction of ${\rm{NO}}_3^ - $ to ${\rm{NO}}_2^ - $ (33), because supplementation with sodium nitrite achieved robust increases in both plasma ${\rm{NO}}_3^ - $ (117 ± 62 µM compared with 41 ± 15 µM, *P* < 0.05) and plasma ${\rm{NO}}_2^ - $ (10.7 ± 10.5 μM compared with 1.7 ± 0.8 μM, *P* < 0.05) concentrations. These results are comparable with what has been reported previously in mice (28), rats (24), and humans (6) in response to sodium nitrate incorporation into the diet.

### The effect of sodium nitrate or nitrite supplementation on murine mitochondrial protein expression and phosphorylation status

**FIGURE 3:**
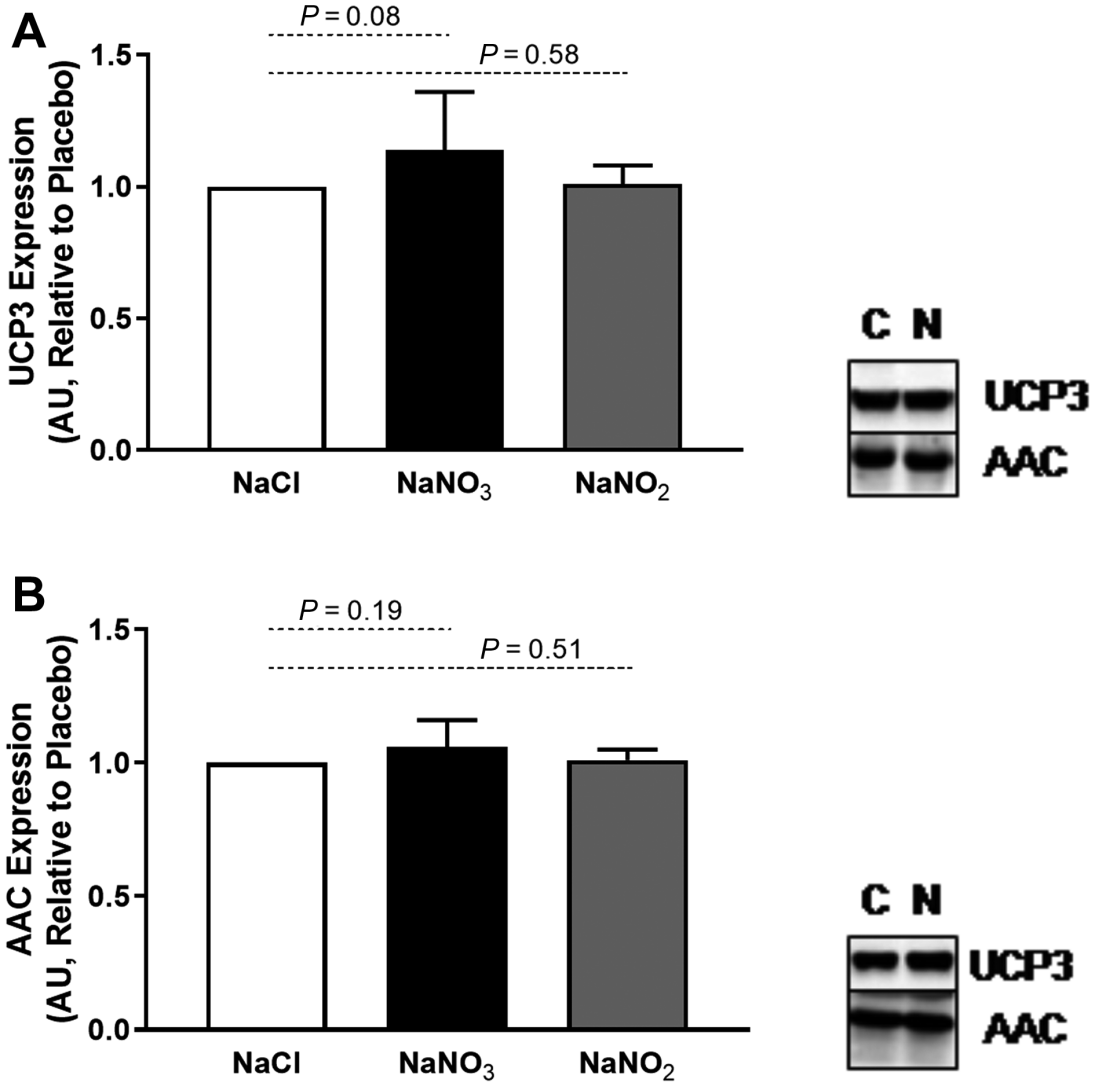
Expression of UCP3 and AAC proteins in mouse skeletal muscle after 7 d dietary supplementation with sodium nitrate (*n* = 12 per group) or sodium nitrite (*n* = 7–8 per group). (A) UCP3 and (B) AAC expression were unchanged by sodium nitrate or sodium nitrite supplementation. AAC, ADP/ATP carrier protein; AU, arbitrary units; C, control; N, nitrate/nitrite; UCP, uncoupling protein.

The mitochondrial proteins AAC (also known as the adenine nucleotide translocases) and UCP have been implicated in the dissipation of mitochondrial membrane potential by facilitating futile proton leak (34, 35). Sodium nitrate supplementation has been reported to improve mitochondrial energetic efficiency through decreased expression of AAC and UCP3, in particular (6). However, when investigated we observed no alterations in expression of these proteins in skeletal muscle in response to supplementation with either sodium nitrate or sodium nitrite (**Figure 3**).

**FIGURE 4:**
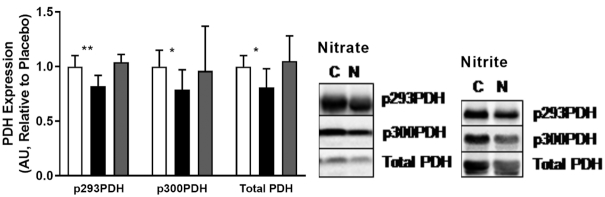
Protein expression and phosphorylation status of PDH in mouse skeletal muscle in response to dietary sodium nitrate (*n* = 12 per group) or sodium nitrite (*n* = 8 per group) supplementation. Sodium nitrate, but not sodium nitrite, supplementation resulted in dephosphorylation of serines 293 and 300 of the PDH complex in mouse skeletal muscle. ^*,**^Significant differences: *P < 0.01, **P < 0.001, compared with control. White bars, NaCl; black bars, NaNO_3_; gray bars, NaNO_2_. AU, arbitrary units; C, control; N, nitrate/nitrite; PDH, pyruvate dehydrogenase.

Sodium nitrite supplementation has also been reported to attenuate fatty acid utilization by mitochondria (27). By extension and according to the Randle cycle (25), a reciprocal boost in glucose utilization and PDH complex activity may be expected to occur. Activity through the PDH complex is regulated through phosphorylation of 3 distinct serine residues (serines 232, 293, and 300), with decreased phosphorylation of these sites corresponding to increased PDH activity. The effects of sodium nitrate or nitrite supplementation in mice on PDH phosphorylation status in skeletal muscle are summarized in **Figure 4**. No change in phosphorylation was detected with sodium nitrite supplementation, whereas only a small change was observed with sodium nitrate (Figure 4). These findings correlate with no observable difference in PDH activity in response to nitrite (**Supplemental Figure 1;**please see**Supplemental Figure 2**for example blots).

### The effect of sodium nitrate or nitrite supplementation on murine mitochondrial energetics

**FIGURE 5:**
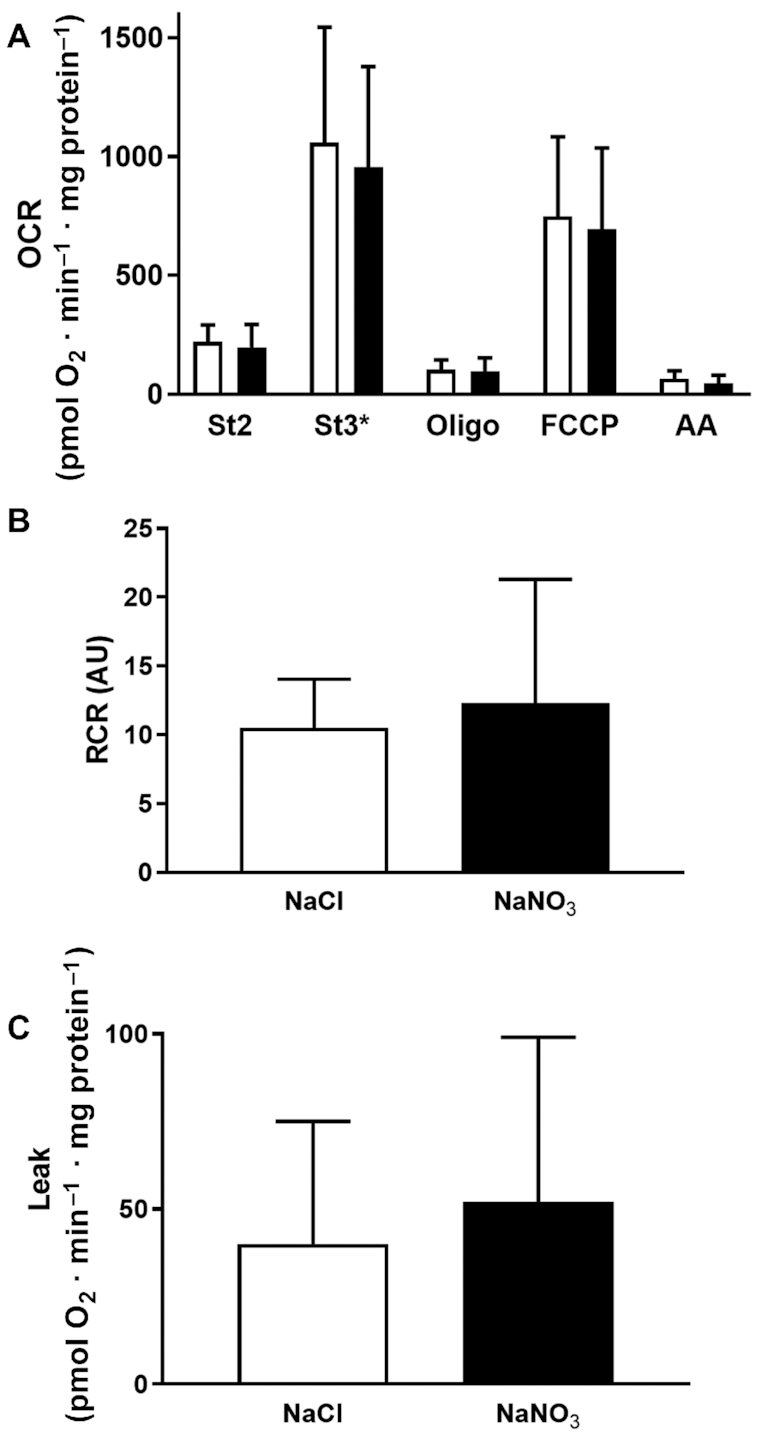
Respiratory activity of skeletal muscle mitochondria isolated from mice supplemented with sodium nitrate (*n* = 9 per group) for 7 d, as assessed by Seahorse XF24 analyzer. (A) Skeletal muscle mitochondrial respiration in response to dietary supplementation with sodium nitrate. (B) RCRs were unchanged in response to supplementation with sodium nitrate. (C) Mitochondrial leak was unchanged in response to sodium nitrate. No significant differences were found between treatment and control groups (at *P* < 0.05; independent *t* test). See the Methods section for further details. White bars, NaCl; black bars, NaNO_3_. AA, antimycin A; AU, arbitrary units; FCCP, carbonyl cyanide-p-trifluoromethoxyphenylhydrazone; OCR, oxygen consumption rate; Oligo, oligomycin; RCR, respiratory control ratio; St2, state 2; St3*, state 3 (ADP at saturated concentrations).

**FIGURE 6:**
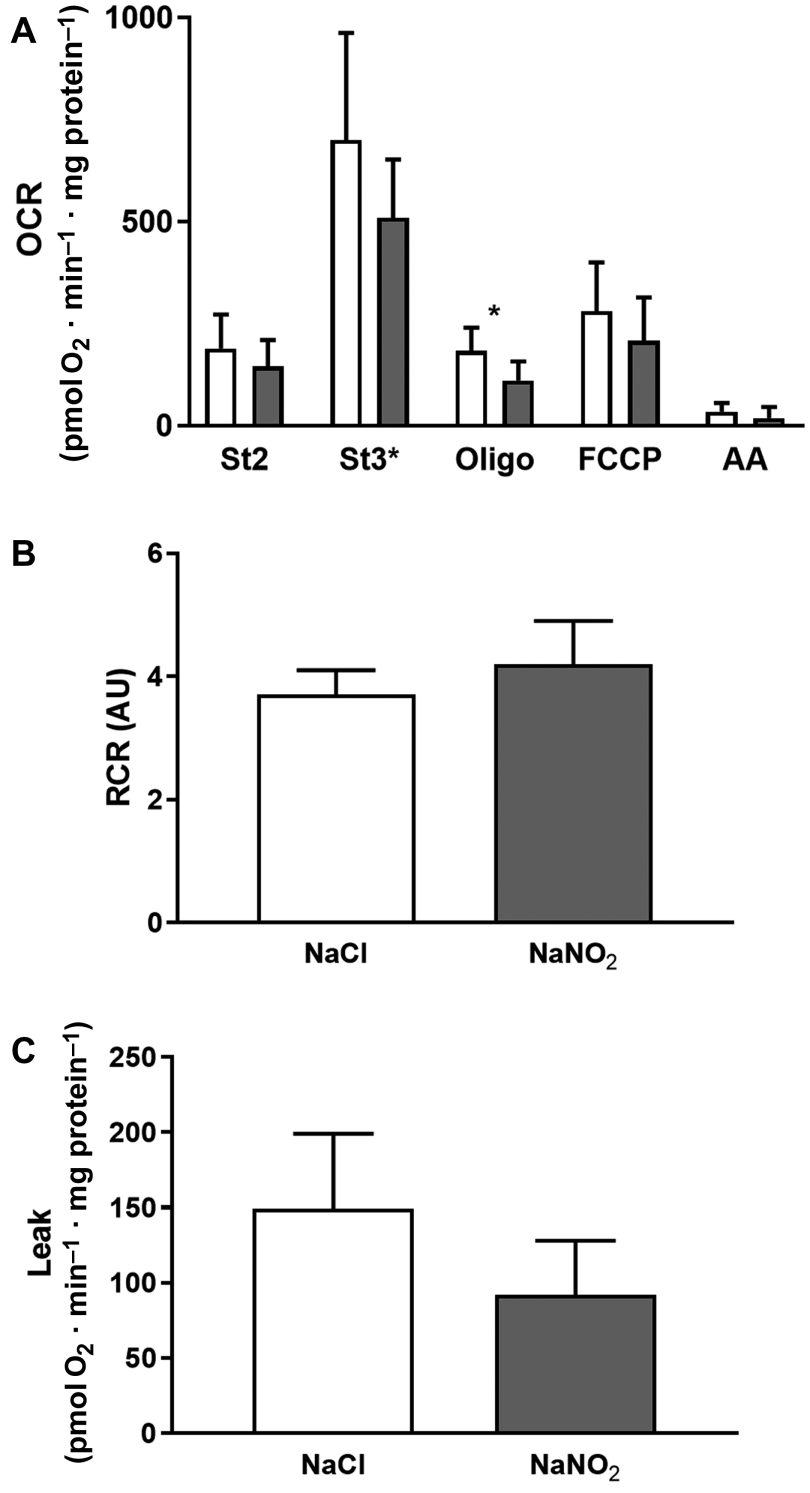
Respiratory activity of skeletal muscle mitochondria isolated from mice supplemented with sodium nitrite (*n* = 5–6 per group) for 7 d, as assessed by Seahorse XF24 analyzer. (A) Skeletal muscle mitochondrial respiration in response to dietary supplementation with sodium nitrite. Nitrite supplementation resulted in decreased oligomycin-induced respiration when compared with control. *Significant differences: *P < 0.05. (B) RCRs were unchanged in response to supplementation with sodium nitrite. (C) Mitochondrial leak was unchanged in response to sodium nitrite. No significant differences were found between treatment and control groups (at *P* < 0.05; independent *t* test). See the Methods section for further details. White bars, NaCl; gray bars, NaNO_2_. AA, antimycin A; AU, arbitrary units; FCCP, carbonyl cyanide-p-trifluoromethoxyphenylhydrazone; OCR, oxygen consumption rate; Oligo, oligomycin; RCR, respiratory control ratio; St2, state 2; St3*, state 3 (ADP at saturated concentrations).

**FIGURE 7:**
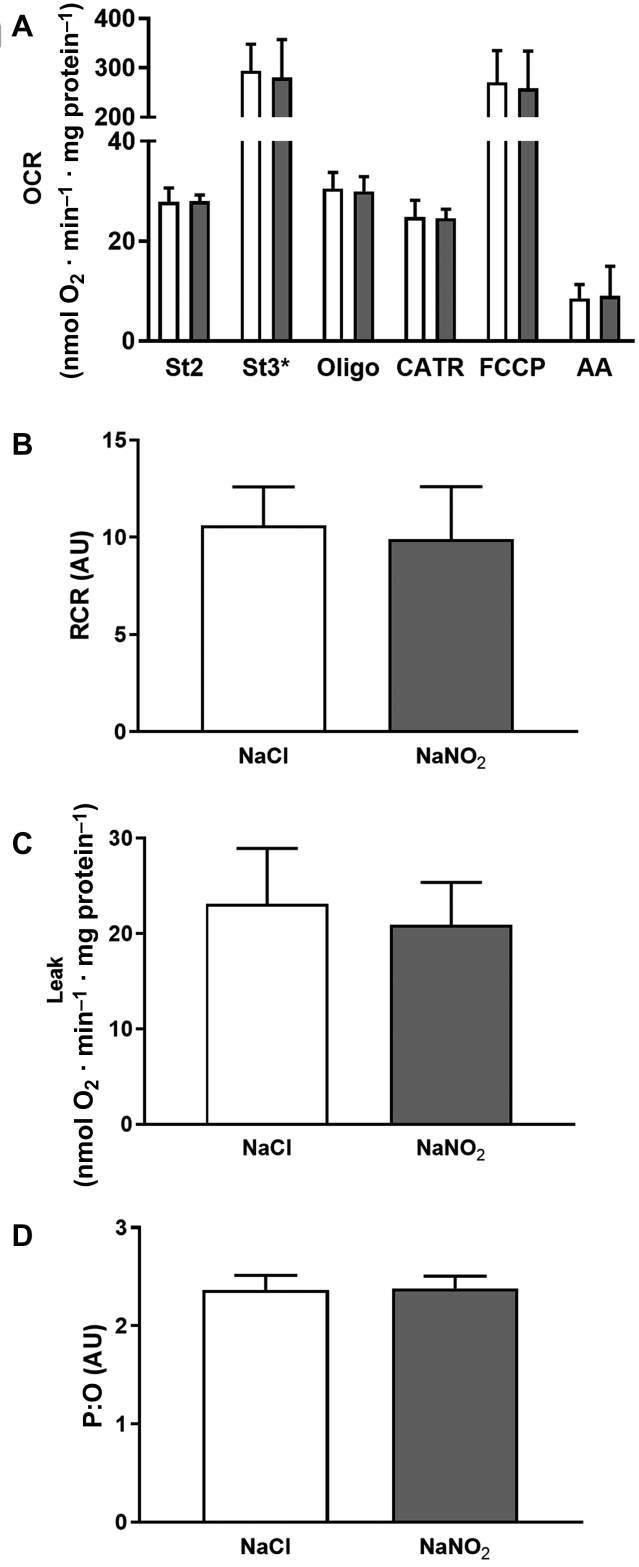
Respiratory activity of skeletal muscle mitochondria isolated from mice supplemented with sodium nitrite (*n* = 8 per group) for 7 d, as evaluated by Clark-type oxygen electrode. (A) Skeletal muscle mitochondrial respiration in response to dietary supplementation with sodium nitrite. (B) RCRs were unchanged in response to supplementation with sodium nitrite. (C) Mitochondrial leak was unchanged in response to sodium nitrite. (D) Mitochondrial P:O ratios were unchanged in response to sodium nitrite. No significant differences were found between treatment and control groups (at *P* < 0.05; independent *t* test). See the Methods section for further details. White bars, NaCl; gray bars, NaNO_2_. AA, antimycin A; AU, arbitrary units; CATR, carboxyatractyloside; FCCP, carbonyl cyanide-p-trifluoromethoxyphenylhydrazone; OCR, oxygen consumption rate; Oligo, oligomycin; P:O, phosphate:oxygen; RCR, respiratory control ratio; St2, state 2; St3*, state 3 (ADP at saturated concentrations).

After observing limited change in protein expression levels, we investigated the impact of both nitrite and nitrate supplementation on mitochondrial efficiency directly by respiratory analysis of isolated mitochondria. As summarized in **Figure 5**, sodium nitrate supplementation had no effect on mitochondrial respiration associated with proton leak activity (OCRs in the presence of oligomycin; Figure 5A, C), coupling efficiency (e.g., the respiratory control ratio; Figure 5B), or respiratory activity in any of the conditions tested. In contrast, sodium nitrite supplementation appeared to decrease mitochondrial activity in the presence of oligomycin (**Figure 6**). However, the respiratory control ratio (Figure 6B) remained unchanged, suggesting that the apparent decrease was due to a general decrease in respiratory activity in all conditions, and was not specific to proton leak (Figure 6C). Further detailed analysis at higher resolution using a Clark-type oxygen electrode in follow-up experiments confirmed that neither proton leak activity (**Figure 7**A, C), the respiratory control ratio (Figure 7B), nor P:O ratio estimates of mitochondrial efficiency (ATP generated per oxygen consumed; Figure 7D) had changed in response to sodium nitrite supplementation. In contrast to previous reports (6, 14), therefore, these results suggest that sodium nitrate and nitrite do not alter mitochondrial proton leak or coupling efficiency to affect physiology.

### Effects of sodium nitrite supplementation on human mitochondrial protein expression

**FIGURE 8:**
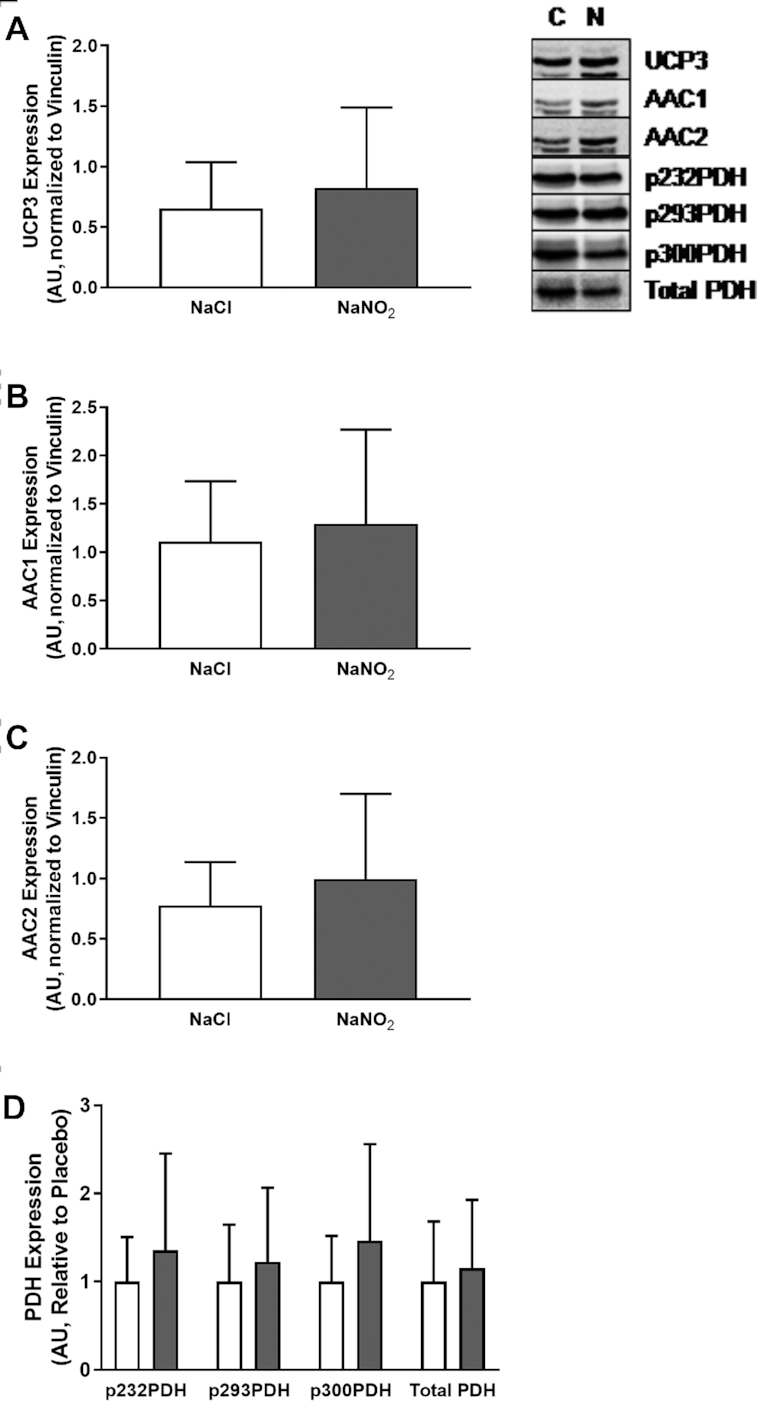
Expression of UCP3 and AAC in human skeletal muscle after infusion of sodium nitrite (*n* = 20) or placebo (*n* = 15). (A) Expression of UCP3 was unchanged in human skeletal muscle in response to sodium nitrite. (B) Expression of AAC1 was unchanged in human skeletal muscle in response to sodium nitrite. (C) Expression of AAC2 was unchanged in human skeletal muscle in response to sodium nitrite. (D) Expression and phosphorylation status of PDH were unchanged in human skeletal muscle in response to sodium nitrite. White bars, NaCl; gray bars, NaNO_2_. AAC, ADP/ATP carrier protein; AU, arbitrary units; C, control; N, nitrate/nitrite; PDH, pyruvate dehydrogenase; UCP, uncoupling protein.

Given our observations with mice, we took the opportunity to investigate the impact of sodium nitrite infusion on expression of mitochondrial UCPs in human skeletal muscle of patients scheduled for CABG surgery (**Figure 8**). For data on the effect of sodium nitrite infusion on cardiac expression of mitochondrial proteins, please see **Supplemental Figures 3** and **4**.

**FIGURE 9:**
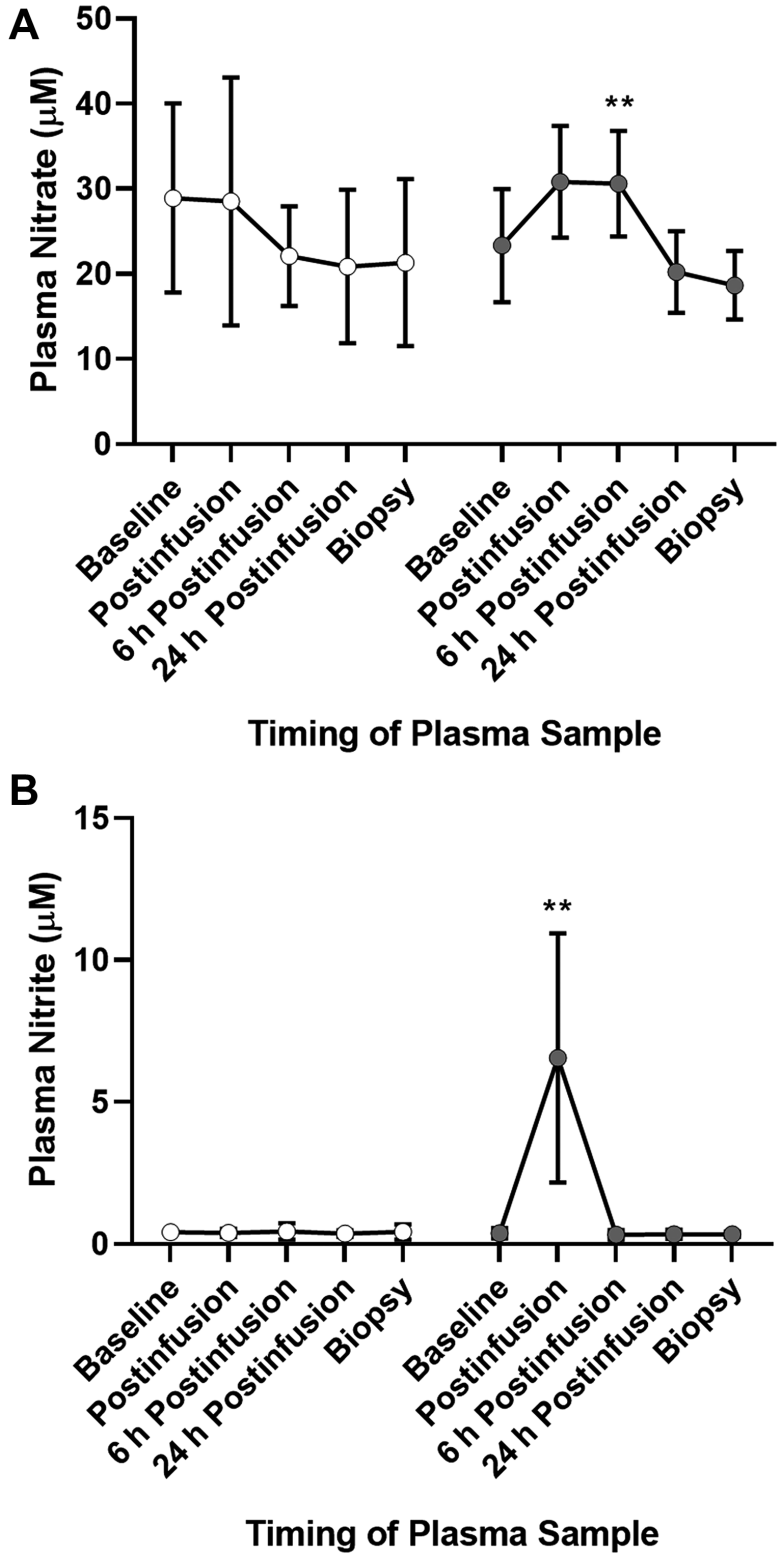
Plasma concentrations of ${\rm{NO}}_3^ - $ and ${\rm{NO}}_2^ - $ in humans over time in response to a 30-min bolus injection of sodium nitrite or saline placebo (see the Methods section for details). (A) Plasma ${\rm{NO}}_3^ - $ concentrations were significantly increased 6 h postinfusion in patients receiving intravenous sodium nitrite (*F*[1,18] = 9.44). **Significant differences: **P < 0.01. (B) Plasma ${\rm{NO}}_2^ - $ concentrations were significantly increased immediately postinfusion in patients receiving intravenous sodium nitrite (*F*[1,32] = 27.342). **Significant differences: **P < 0.01. White circles, NaCl; gray circles, NaNO_2_.

The clinical characteristics of this cohort are summarized in **Table 1**, whereas pharmacokinetics of plasma ${\rm{NO}}_3^ - $ and ${\rm{NO}}_2^ - $ in response to infusion are depicted in **Figure 9**. Infusion of sodium nitrite resulted in an immediate increase in plasma ${\rm{NO}}_2^ - $ concentrations, whereas plasma ${\rm{NO}}_3^ - $ concentrations had increased by 6 h postinfusion.

**TABLE 1:** Clinical characteristics of the human study cohort^1^

	Placebo (*n* = 15)	Nitrite (*n* = 20)	*P*
Age, y	67 ± 10	62 ± 9	0.199
Sex, male	11 (73.3)	19 (95.0)	0.070
Height, cm	167 ± 10	172 ± 10	0.130
Weight, kg	89 ± 15	88 ± 16	0.959
Body surface area, m^2^	2.02 ± 0.22	2.04 ± 0.23	0.714
SBP baseline, mm Hg	131 ± 17	125 ± 19	0.356
SBP postinfusion, mm Hg	123 ± 22	117 ± 21	0.467
DBP baseline, mm Hg	76 ± 9	73 ± 10	0.383
DBP postinfusion, mm Hg	70 ± 9	68 ± 12	0.592
HR baseline, bpm	61 (56, 68)	64 (57, 72)	0.227
HR postinfusion, bpm	58 (55, 63)	64 (55, 74)	0.178
Diabetes mellitus	4 (26.7)	7 (35.0)	0.599
Myocardial infarction	7 (46.7)	7 (35.0)	0.486
Hypertension	11 (73.3)	10 (50.0)	0.163
Smoking status			0.325
Never	5 (33.3)	10 (50.0)	
Ex-smoker	9 (60.0)	7 (35.0)	
Current	1 (6.7)	3 (15.0)	
Aspirin	12 (80.0)	13 (65.0)	0.331
β-blockers	14 (93.3)	16 (80.0)	0.265
Oral nitrates	2 (13.3)	4 (20.0)	0.605
Calcium channel blockers	4 (26.7)	4 (20.0)	0.642
ACEi/ARB	8 (53.3)	12 (60.0)	0.693
Statins	15 (100.0)	19 (95.0)	0.380

^1^Values are *n* (%), mean ± SD, or median (IQR). ACEi, angiotensin-converting enzyme inhibitors; ARB, angiotensin receptor type 2 blockers; DBP, diastolic blood pressure; HR, heart rate; SBP, systolic blood pressure.

Infusion of sodium nitrite before surgery did not alter the expression of UCP3, AAC1, or AAC2 in human skeletal muscle. Similarly, PDH expression and phosphorylation status remained unchanged. These findings in humans are consistent with our findings in mice, where little or no change in these proteins was observed.

## Discussion

Numerous studies have reported on improved exercise performance in healthy participants in response to ingestion of inorganic nitrate (4, 5, 7, 13, 14, 36). The inclusion of inorganic nitrate into the diet, whether through dietary sources (e.g., beetroot, spinach) or through other therapeutic avenues (e.g., supplements), has been consistently reported to improve muscle performance (8, 13, 37) and to decrease the oxygen cost of submaximal exercise (4, 5, 7, 14), with no detectable changes in plasma substrate concentrations (7, 14, 36). These improvements in exercise performance have also been observed in cardiovascular diseases such as stable angina (38), and heart failure with either preserved (9) or reduced (10) ejection fraction.

Based in part on data obtained via near infrared spectroscopy (NIRS), as well as unchanged plasma substrate concentrations, it was postulated that the exercise benefits of inorganic nitrate could be attributed to improvements in mitochondrial efficiency, specifically increased coupling of mitochondrial respiration to ATP generation, through decreased expression of AAC and UCP3 and reduced proton leak activity (6). Given the canonical understanding of the entero-salivary circuit for absorption of exogenous nitrates (39, 40), together with the observations regarding NO and mitochondrial respiration (41), these findings provide an attractive mechanism to account for the apparent exercise benefits of inorganic nitrates.

It should be noted that studies using the NIRS technique have shown reduced muscle oxygen consumption after ingestion of inorganic nitrate (4), but this is directly related to alterations in regional blood flow (42, 43). This is particularly relevant when considering the known vasodilator properties of nitrite, the putative active component of inorganic nitrate (1, 15, 44-46). Using quantitative fMRI, reductions in muscle oxygen consumption at submaximal exercise with inorganic nitrate were shown to be more prominent in muscles with higher proportions of type 1 fibers, suggesting a mitochondrial, rather than vascular, effect (47). However, previous human studies have observed that oral inorganic nitrate does not alter glucose uptake nor insulin sensitivity (36).

Importantly, our investigation failed to find any alterations in mitochondrial respiration in response to inorganic nitrite, nor were any changes in expression of the proteins AAC1, AAC2, or UCP3 observed in either human or murine skeletal muscle tissue. Indeed, recent work by others has also cast doubt on the idea that inorganic nitrate improves mitochondrial respiratory efficiency: whereas there was a slight reduction in whole body oxygen consumption, Whitfield et al. (23) observed no changes in mitochondrial respiration or UCP content in response to inorganic nitrate (ingested as beetroot juice) (23). Furthermore, we found no substantive changes in the phosphorylation status of the PDH complex, nor any increase in PDH activity. As such, there is now a growing body of evidence to indicate that previously observed exercise benefits in response to inorganic nitrate/nitrite are unlikely due to improvements in mitochondrial oxygen efficiency.

There are some limitations to the present study which need to be acknowledged. Although broadly similar, plasma ${\rm{NO}}_3^ - $ and ${\rm{NO}}_2^ - $ concentrations in our murine cohorts were slightly higher than what has been reported previously (28). In addition, we collected no functional data to complement our physiological data. With regards to our human data, plasma ${\rm{NO}}_3^ - $ and ${\rm{NO}}_2^ - $ concentrations at the time of biopsy were similar to those at baseline, although this does not preclude the possibility of long-lasting effects: indeed, we have recently reported on a persulfide-signaling-based mechanism whereby sodium nitrite may induce long-lasting effects in vascular tissue (48). Lastly, this investigation does not address the possibility of divergent effects between dietary nitrate and dietary nitrite supplementation: dietary nitrate supplementation was observed to reduce whole body oxygen consumption (while leaving the respiratory exchange ratio and indexes of glucose handling unchanged), yet infusion with sodium nitrite failed to demonstrate the same effects (36). These results were replicated to an extent in mice and humans (23, 24), where supplementation with nitrate-rich beetroot juice or sodium nitrate solution also reduced whole body oxygen consumption. These latter studies reported similar results to our own in that they did not observe any reductions in mitochondrial leak nor improvements in mitochondrial coupling efficiency.

The evidence underlying the vascular effects of inorganic nitrate/nitrite remains convincing, however. Hypoxic potentiation of inorganic nitrite as a vasodilator, and its augmentation in the presence of hemoglobin, have been reported previously (15), and similar work has reported a seeming dependence on myoglobin for the vascular effects of nitrite (49). In a clinical study involving chronic HFrEF patients, nitrite infusion resulted in decreases in pulmonary vascular resistance and right atrial pressure, as well as increased stroke volume, correlating with increased estimated transseptal gradient (defined as pulmonary capillary wedge pressure minus right atrial pressure), with alleviation of diastolic ventricular interaction suggested as a likely mechanism (45). Similar effects have also been observed in response to inhaled nitrite in HFpEF patients (50, 51). Elucidation of the mechanisms underlying the vascular effects of nitrite, however, is beyond the scope of the present investigation.

The therapeutic benefits of inorganic nitrate/nitrite are unlikely to be due to direct modulation of skeletal muscle mitochondrial metabolism. These results do not preclude that the underlying benefits of inorganic nitrite may be mediated through vascular effects: indeed, modulation of vascular function may even account for previous reports of altered metabolism in response to inorganic nitrate/nitrite.

## Supplementary Material

nqz245_Supplemental_File

## Notes

Supported by Medical Research Council program grant MRC G1001340 (to M Madhani, M Feelisch, and MP Frenneaux).

Supplemental Figures 1–4 are available from the “Supplementary data” link in the online posting of the article and from the same link in the online table of contents at https://academic.oup.com/ajcn/.

MN and NEKP contributed equally to this work.

Data described in the manuscript, code book, and analytic code will be made available upon request pending application and approval.

Abbreviations used: AAC, ADP/ATP carrier protein; CABG, coronary artery bypass graft; FCCP, carbonyl cyanide-p-trifluoromethoxyphenylhydrazone; HFpEF, heart failure with preserved ejection fraction; HFrEF, heart failure with reduced ejection fraction; MAS, mitochondrial assay solution; NIRS, near infrared spectroscopy; NO, nitric oxide; OCR, oxygen consumption rate; PDH, pyruvate dehydrogenase; P:O, phosphate:oxygen; PVDF, polyvinylidene difluoride; TBS, Tris-buffered saline; UCP, uncoupling protein.
