# Supplementary material for: Inorganic nitrate and nitrite supplementation fails to improve skeletal muscle mitochondrial efficiency in mice and humans
Source: Am J Clin Nutr. 2019 Oct 10;111(1):79–89. doi: 10.1093/ajcn/nqz245 (PMC6944528; doi:10.1093/ajcn/nqz245)

**Supplementary Figure 1**: Pyruvate dehydrogenase activity (as reflected by the rate of acetyl coenzyme A accumulation) was unchanged in murine skeletal muscle in response to nitrite.

**Supplementary Figure 2**: **A**, Example blots of expression of uncoupling proteins in mouse skeletal muscle following treatment with sodium nitrate. **B**, Example blots of expression of uncoupling proteins in murine skeletal muscle following treatment with sodium nitrite. **C**, Example blots of expression and phosphorylation status of PDH in mouse skeletal muscle following treatment with sodium nitrate. **D**, Example blots of expression and phosphorylation status of PDH in mouse skeletal muscle following treatment with sodium nitrite. **E**, Example blots of expression of uncoupling proteins in human skeletal muscle following treatment with sodium nitrite. **F**, Example blots of expression of and phosphorylation status of PDH in human skeletal muscle following treatment with sodium nitrite.

**Supplementary Figure 3**: Expression of UCP3 and AAC proteins in human right atrial appendage following infusion of sodium nitrite (n=14) or placebo (n=19). **A**, Expression of UCP3 protein was unchanged in human skeletal muscle in response to sodium nitrite. **B**, Expression of AAC1 was unchanged in human skeletal muscle in response to sodium nitrite. **C**, Expression of AAC2 was unchanged in human skeletal muscle in response to sodium nitrite. **D**, Expression and phosphorylation status of PDH was unchanged in human skeletal muscle in response to sodium nitrite. Note: = NaCl, = NaNO_2_.

**Supplementary Figure 4**: Expression of UCP3 and AAC proteins in human left ventricle following infusion of sodium nitrite (n=15) or placebo (n=10). **A**, Expression of UCP3 protein was unchanged in human skeletal muscle in response to sodium nitrite. **B**, Expression of AAC1 was unchanged in human skeletal muscle in response to sodium nitrite. **C**, Expression of AAC2 was unchanged in human skeletal muscle in response to sodium nitrite. **D**, Expression and phosphorylation status of PDH was unchanged in human skeletal muscle in response to sodium nitrite. Note: = NaCl, = NaNO_2_.

**Supplementary Figure 1**

**Supplementary Figure 2**

**Supplementary Figure 3**


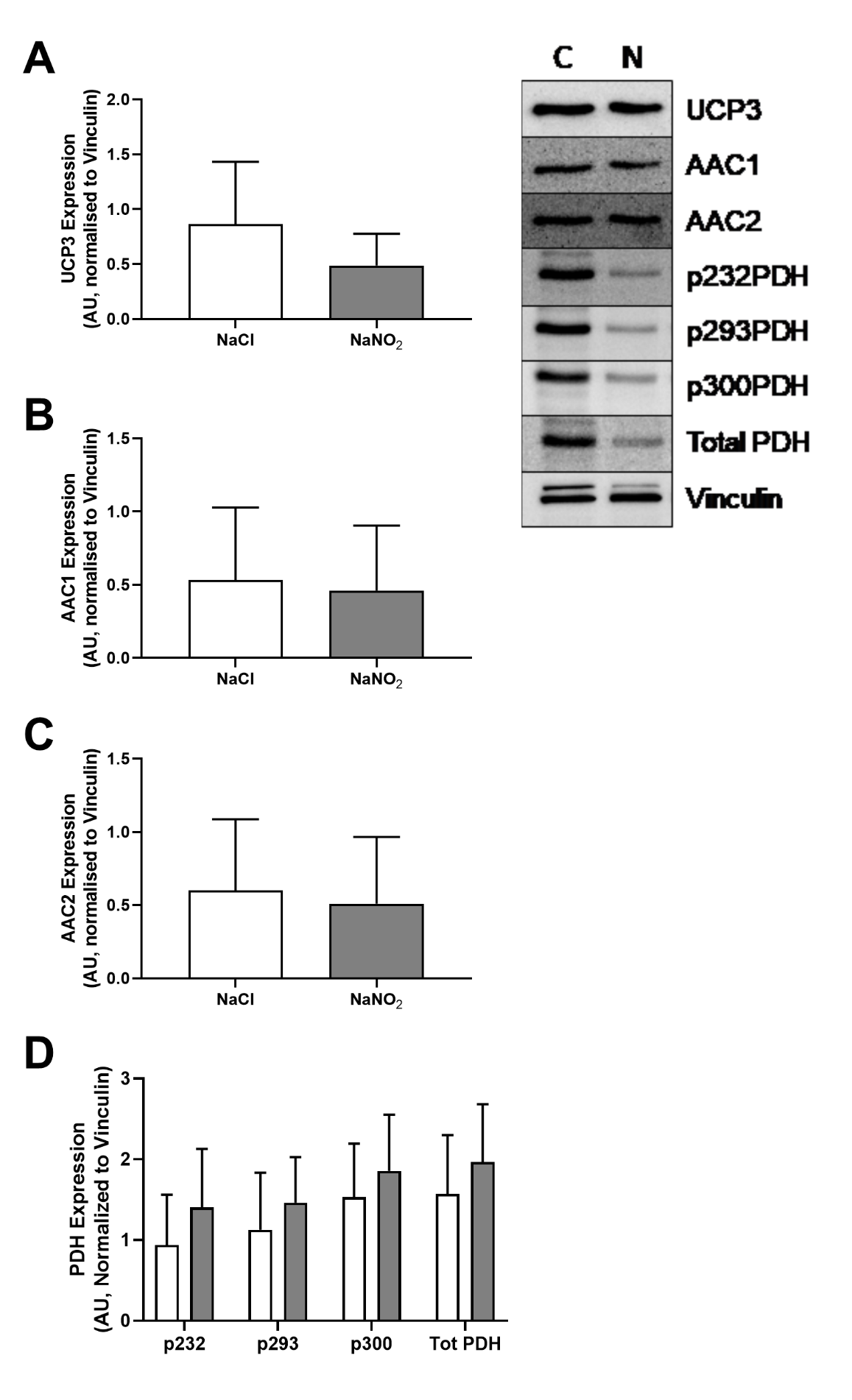


**Supplementary Figure 4**


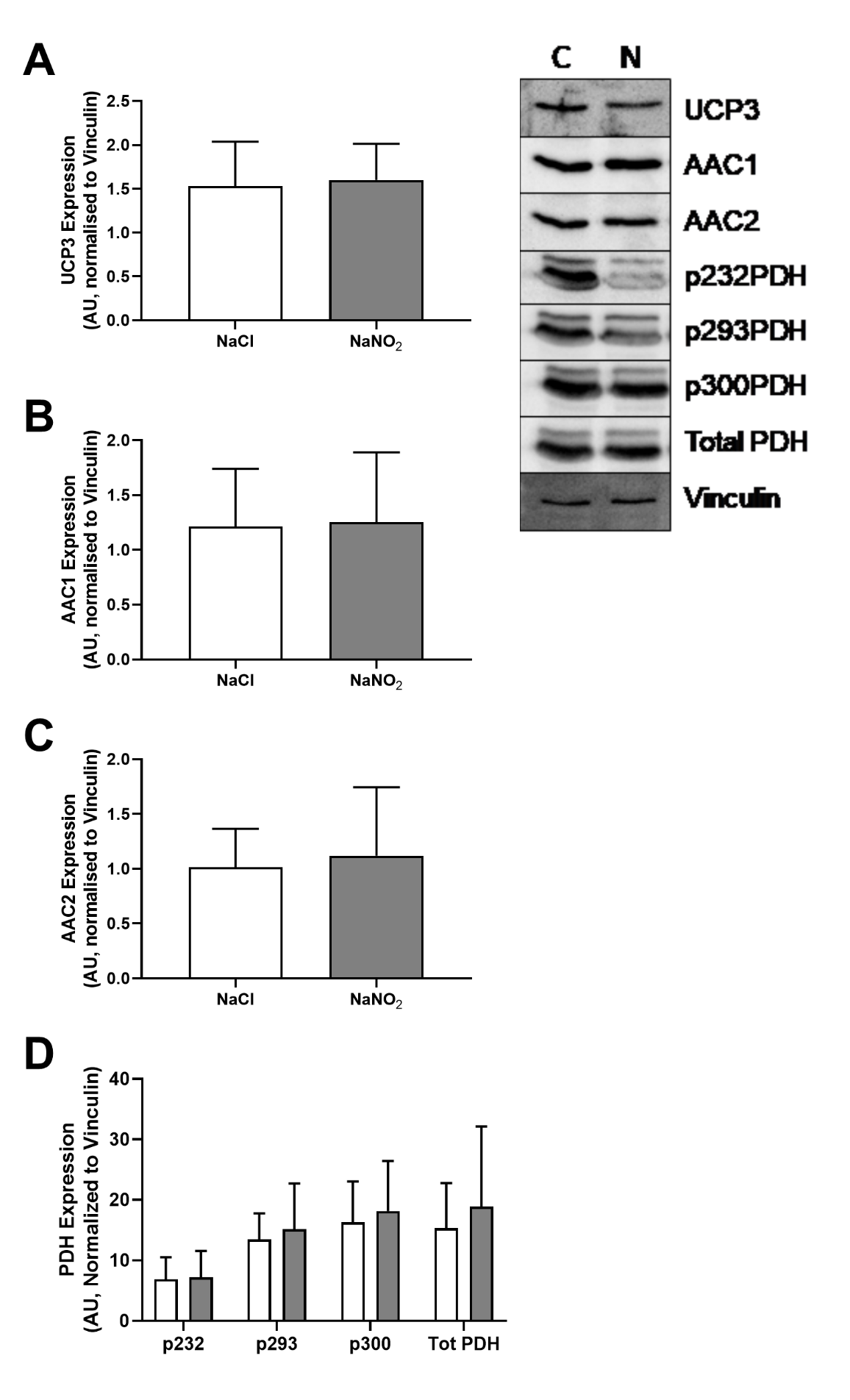

Supplement: nqz245_Supplemental_File [file nqz245_supplemental_file.docx]
